# Pilon: An Integrated Tool for Comprehensive Microbial Variant Detection and Genome Assembly Improvement

**DOI:** 10.1371/journal.pone.0112963

**Published:** 2014-11-19

**Authors:** Bruce J. Walker, Thomas Abeel, Terrance Shea, Margaret Priest, Amr Abouelliel, Sharadha Sakthikumar, Christina A. Cuomo, Qiandong Zeng, Jennifer Wortman, Sarah K. Young, Ashlee M. Earl

**Affiliations:** 1 Broad Institute of MIT and Harvard, Cambridge, Massachusetts, United States of America; 2 VIB Department of Plant Systems Biology, Ghent University, Ghent, Belgium; The University of Hong Kong, Hong Kong

## Abstract

Advances in modern sequencing technologies allow us to generate sufficient data to analyze hundreds of bacterial genomes from a single machine in a single day. This potential for sequencing massive numbers of genomes calls for fully automated methods to produce high-quality assemblies and variant calls. We introduce Pilon, a fully automated, all-in-one tool for correcting draft assemblies and calling sequence variants of multiple sizes, including very large insertions and deletions. Pilon works with many types of sequence data, but is particularly strong when supplied with paired end data from two Illumina libraries with small *e.g.*, 180 bp and large *e.g.*, 3–5 Kb inserts. Pilon significantly improves draft genome assemblies by correcting bases, fixing mis-assemblies and filling gaps. For both haploid and diploid genomes, Pilon produces more contiguous genomes with fewer errors, enabling identification of more biologically relevant genes. Furthermore, Pilon identifies small variants with high accuracy as compared to state-of-the-art tools and is unique in its ability to accurately identify large sequence variants including duplications and resolve large insertions. Pilon is being used to improve the assemblies of thousands of new genomes and to identify variants from thousands of clinically relevant bacterial strains. Pilon is freely available as open source software.

## Introduction

Massively parallel sequencing technology has dramatically reduced the cost of genome sequencing, making the generation of large numbers of microbial genomes accessible to a wide range of biological researchers. For example, a single Illumina HiSeq2500 has the ability to generate the equivalent of 300 bacterial genomes of sequencing data in a single day using only one flow cell. Comparisons of whole genome sequence data from hundreds of microorganisms have provided unprecedented views on all aspects of microbial diversity, and there is growing recognition that ‘hundreds’ of genomes is the minimum scale needed to address pressing questions related to microbial evolution, diversity, pathogenicity and resistance to antimicrobial drugs [1]–[4]. As such, the methods needed to analyze these large volumes of data — including assembling and calling variants relative to a reference — must be robust, accurate, scalable, and able to operate without human intervention.

Several computational methods exist that make improvements to the quality of draft assemblies by recognizing and correcting errors involving (i) single bases and small insertion/deletion events (indels) [5], (ii) gaps [6], (iii) read alignment discontinuities [7] or by reconciling multiple *de novo* assemblies into an improved consensus assembly [8]. However, no single tool performs integrated assembly improvement of all error types. Computational tools for identifying sequence polymorphisms also exist [9], [10], but focus primarily on identifying variants in the human genome [11], and particularly small events (SNPs and small indels) or structural rearrangements (chromosomal rearrangements) [11]. Furthermore, many of these tools require multiple steps to identify and subsequently filter variants to remove noise and false calls. In addition, for tools able to identify variants that exceed the length of the sequence reads (read-length) being evaluated, they generally indicate the approximate chromosomal location and estimated size of the predicted variant relative to the reference, but often do not provide exact coordinates [11]. For insertions that are longer than the read-length - particularly common in the microbial world - current tools do not assemble and report the inserted sequence.

**Figure 1:**
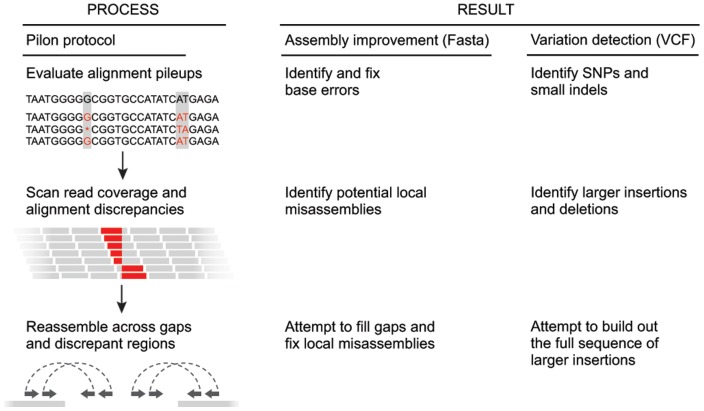
Simplified overview of the Pilon workflow for assembly improvement and variant detection. The left column depicts the conceptual steps of the Pilon process, and the center and right columns describe what Pilon does at each step while in assembly improvement and variant detection modes, respectively. During the first step (top row), Pilon scans the read alignments for evidence where the sequencing data disagree with the input genome and makes corrections to small errors and detects small variants. During the second step (second row), Pilon looks for coverage and alignment discrepancies to identify potential mis-assemblies and larger variants. Finally (bottom row), Pilon uses reads and mate pairs which are anchored to the flanks of discrepant regions and gaps in the input genome to reassemble the area, attempting to fill in the true sequence including large insertions. The resulting output is an improved assembly and/or a VCF file of variants.

We introduce Pilon, an integrated software tool for comprehensive microbial genome assembly improvement and variant detection, including detection of variants that exceed sequence read-length. Conceptually, Pilon treats assembly improvement and variant detection as the same process (Figure 1). Both start with an input genome — either an existing draft assembly or a reference assembly from another strain — and use evidence from read alignments to identify specific differences from the input genome supported by the sequencing data. Applying those changes to a draft genome assembly yields an improved assembly, while reporting the changes with respect to a reference genome yields variant calls.

In genomic regions where read alignments are poor, Pilon is capable of filling out and correcting sequence through an internal local reassembly process. This capability allows Pilon to further improve assemblies by filling gaps and correcting local mis-assemblies, and it also enables Pilon to capture many large insertion, deletion, and block substitution variants in their entirety. These larger events are often completely missed or inaccurately characterized by conventional variant calling tools that rely solely on read alignments. Pilon has built-in heuristics to determine which corrections and calls are of high confidence, so no separate filtering criteria need be specified. This allows for the automated processing of hundreds or thousands of data sets representing different microbial species with minimal human intervention.

We benchmarked Pilon both as an assembly refinement tool and variant caller. For assembly refinement, we used finished reference genome sequences from *Mycobacterium tuberculosis* F11, *Streptococcus pneumoniae* TIGR4 and *Candida albicans* SC5314 as benchmarks to evaluate the accuracy of Pilon in improving draft assemblies. Pilon-improved assemblies were more contiguous and complete than non-Pilon-improved assemblies and contained improved sequences for genes implicated in pathogen-host interaction and virulence. We also evaluated Pilon's performance against tools specializing in assembly base quality improvement and gap filling, and, in each case, Pilon made more correct improvements while making far fewer mistakes than the other tools. For variant calling, we used read data from *M. tuberculosis* F11 to call polymorphisms against the finished *M. tuberculosis* H37Rv genome to evaluate Pilon's ability to accurately call polymorphisms. Pilon performed as well or better when compared with two state-of-the-art variant detection tools in calling small variants, and Pilon differentiated itself in its ability to identify large-scale variants.

## Results

### Assembly improvement evaluation

#### Assessing accuracy on bacterial assemblies

To test the accuracy of Pilon's improvements on bacterial assemblies, we sequenced and created draft assemblies for two bacterial strains with finished references: *S. pneumoniae* TIGR4 and *M. tuberculosis* F11 (see Methods). These strains were chosen because they represent different GC content (40% and 66% GC content, respectively) and both possess genomic features that are known to confound assemblers, leading to mis-assembled and/or incomplete genome sequences [12]–[14]. Sequence reads from both libraries were aligned back to their respective draft assemblies using *BWA* [15], and Pilon was run with those alignments.

To assess the benefits of running Pilon, we compared the original draft and Pilon-improved assemblies to each other and to their respective finished genome sequence. Pilon made significant improvements to the contiguity of both draft assemblies, increasing the contig N50 size by 443 Kbp for *S. pneumoniae* TIGR4 (see Table 1) and 196 Kbp for *M. tuberculosis* F11, even though the F11 draft assembly had been generated with assistance from a close reference. In addition, Pilon assemblies were more complete, with the *M. tuberculosis* F11 and *S. pneumoniae* TIGR4 Pilon-improved assemblies containing an additional 11,516 bp and 9,608 bp, respectively.

**Table 1:** Summary assembly statistics before and after Pilon improvement.

Genome	*M. tuberculosis F11*	*S. pneumoniae TIGR4*	*C. albicans SC5314*
Contig N50 Increase	196 kb	443 kb	56 kb
Bases Added	11,516	9,608	33,804
Gaps Closed	9	9	54
Gaps Shrunk	7	7	102
Single-base Modifications	20	27	26,939
Mis-assembly fixes	3	1	44

In all cases the assemblies were more contiguous, contained more bases, and had fewer gaps and errors after Pilon improvement.

Observed gains in genome coverage and contig N50 were principally due to Pilon's ability to recognize and fill (or partially fill) by local assembly “captured gaps”, *i.e.*, missing sequence between contigs within a scaffold. When run with default settings, Pilon does not introduce ambiguous bases or additional Ns during this process. Across the two draft assemblies, Pilon completely and accurately filled 17 of the 44 captured gaps (39% closure rate) including 8 gaps that represented more than 1 Kbp in sequence length (see Table S1). None of Pilon's gap closures were incorrect, though one was judged to be “no worse”: the sequence used to bridge the gap was correct, but an error in the original assembly in one of the gap flanks was not detected by Pilon. An additional 14 gaps (32% of total captured gaps) were partially filled by Pilon, and 13 (93%) of those extensions were error-free. The one partial fill judged to be “Incorrect” involved a repetitive structure that Pilon extended into flanking sequence belonging to a different copy of the repeat.

We compared Pilon's ability to close gaps in these assemblies with two other tools commonly used for this purpose, IMAGE [16] and GapFiller [17] (see Table S1b). Pilon's overall gap closure rate was only somewhat higher than that of the other tools, but its accuracy was dramatically better. Across the two assemblies, IMAGE closed 13 captured gaps (30% closure rate) but only two of those closures were found to be correct by alignment with the reference (15% precision). Similarly, GapFiller closed 16 of captured gaps (36% closure rate) in the two assemblies, but only four of its closures were correct (25% precision). In addition to filling captured gaps, Pilon also corrected 43 single-bases and 4 small indels across both genomes, and all 47 changes were found to be correct by alignment against the reference (100% accuracy; see Table S2). By comparison, iCORN [18] made 47 single-base changes and 2 single-base deletions, but only 35 of the 49 (71%) of its changes were correct.

Optionally, Pilon can also make changes to genomic locations at which it finds significant evidence for more than one alternative, choosing the allele with the most support even where the evidence is insufficient to make a confident call. When run with this option on these assemblies, Pilon made 10 changes to ambiguous bases, but only 3 were verified to be correct. This option is turned off by default starting with Pilon version 1.8.

Pilon also detected and attempted to fix local mis-assemblies by reassembling contig regions that were suspected to be incorrectly assembled. Three of these regions were correctly fixed (see Table 1 and Table S1) and a fourth we classified as “No worse”. For the latter, Pilon correctly identified a repetitive region within the original *M. tuberculosis* F11 draft assembly that contained extra sequence with respect to the *M. tuberculosis* F11 reference. However, Pilon's change introduced a deletion with respect to the reference, underscoring the difficulty of accurately assembling repetitive regions with short read data [13].

**Figure 2:**
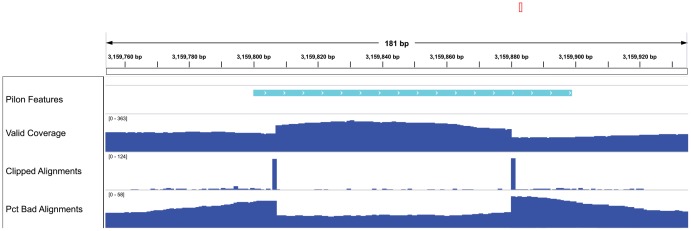
Example Pilon generated genome browser tracks. This region was flagged by Pilon as containing a possible local mis-assembly, but Pilon was unable to determine a fix due to a tandem repeat sequence. The tracks shown here include: *Pilon Features* track indicating the extent of the region flagged by Pilon as containing a potential mis-assembly, *Valid Coverage* track indicating the sequence coverage of valid read pair alignments excluding the clipped portions of the alignments, *Clipped Alignments* track indicating the number of reads soft-clipped at each location, *Pct Bad Alignments* track indicating the percentage of the total reads aligned to each location which are not part of *Valid Coverage*. These tracks are created with the ‘—tracks' command-line option. Together, these tracks reveal the true bounds of the mis-assembly, and indicate that there are likely missing copies of the tandem repeat in the draft assembly. In this case, manual analysis revealed the draft assembly was missing two of three full copies of a 57-base tandem repeat.

For the *M. tuberculosis* F11 and *S. pneumoniae* TIGR4 Pilon improved assemblies, there were 13 and 4 regions, respectively, where Pilon detected a problem in the draft assembly, but was unable to provide solutions. In each of these cases, Pilon flagged the coordinates of the problematic region, and, in 10 of these cases, it also reported the length of the detected tandem repeat confounding resolution of the region. For example, Figure 2 shows scaffold00001 coordinates 3159800–3159898 of the *M. tuberculosis* F11 draft assembly, along with Pilon-generated genome browser tracks representing some of the internal metrics it used to identify this region as problematic. In this case, Pilon noted that it was unable to resolve a 57 bp tandem repeat, which enabled an experienced analyst to confirm the presence of a mis-assembly and accurately narrow the bounds of the unresolvable region. Manual comparison of the draft assembly with the reference revealed that there should have been three full and one partial copies of the 57 bp repeat in tandem, whereas the draft assembly only contained one full and one partial copy of the repeat.

#### Effect of assembly improvements on gene calls

**Figure 3:**
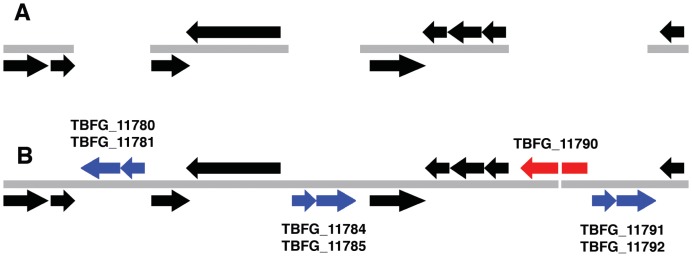
Comparative view of a transposase-rich region of the *M. tuberculosis* F11 genome (coordinates 1,991,000 to 2,006,300) obtained from the draft (A) and Pilon-improved (B) assemblies. In the draft assembly, three regions containing transposases (shown in blue) remained unassembled resulting in gaps. In the Pilon-improved assembly, all three sets of transposases were successfully assembled. The Pilon-improved assembly also contained a hypothetical gene, *TBFG_11790* (shown in red), missing from the draft assembly. Though *TBFG_11790* was not fully closed in the Pilon-improved version, closer inspection revealed that there was a 42 bp overlap in assembled sequence at this site. By default, Pilon will not close gaps unless there is at least 95 bp overlapping sequence to minimize spurious joins.

To assess the impact of Pilon-improvement on gene calls (*i.e.*, functional interpretation of the genome), we examined Pilon improvements with respect to genes by investigating the regions that were affected by Pilon modifications and the effect of these modifications on coding sequences. Thirty-two genes and seven intergenic regions were impacted by Pilon changes to the *M. tuberculosis* F11 Pilon-improved assembly; of these, nearly all (95%; 37 of 39) were correct improvements. Nearly half (13) of the genes that were affected by a fix involved transposases that were completely or partially filled with sequence that perfectly matched the reference genome (see Table S3). One additional transposase had a single base pair corrected with perfect match to the reference. A particularly complex 13 Kbp region in *M. tuberculosis* F11 is highlighted in Figure 3. This region harbors three sets of transposases in close proximity that were not captured in the draft *M. tuberculosis* F11 assembly, but were accurately filled in by Pilon. Two of the gaps were completely closed, and the third transposase set was completely captured along with an additional gene. However, due to Pilon's conservative overlap requirement for closure (95 bp), that gap was not closed despite a 42 bp overlap in the extended flank sequences.

Of the remaining 19 genes, 6 were PE/PPE family protein encoding genes. Five corrections were perfect and, in one case (TBFG_11946), Pilon identified the problematic region, but could not completely resolve the problem. However, the correction that Pilon applied did not make the situation worse. Pilon also identified and accurately corrected a mis-assembly (highlighted in Figure S1) in which a gene had been truncated due to a collapsed repeat in the draft assembly.

In *S. pneumoniae* TIGR4, 20 genes and 12 intergenic regions were affected by fixes from Pilon. A majority (15 of 20) of the improved genes were transposases, of which Pilon was able to completely or partially fill 8 that matched completely and perfectly with the reference; the remaining 7 were individual base pair corrections. Pilon was also able to partially fill other genes encoding repetitive cell wall surface proteins - including choline binding protein A [19] and pneumococcal surface protein A [20] - both implicated in adhesion and virulence in *S. pneumoniae*.

#### Assessing accuracy on the assembly of the larger, polymorphic genome of *C. albicans*

To evaluate Pilon's ability to accurately improve assemblies of diploid genomes containing a high level of heterozygosity, we ran Pilon on an Illumina ALLPATHS-LG assembly of the SC5314 strain of *C. albicans* (Methods), for which there is a high quality reference curated by the Candida Genome Database (www.candidagenome.org). At 14.3 Mb, the *C. albicans* genome is 3- to 7-fold larger than the bacterial genomes evaluated here. It consists of 8 chromosomes that are present at diploid levels with an average of one SNP found at every 330–390 bases, although large regions of most chromosomes display loss of heterozygosity [21], [22].

Pilon was capable of improving the assembly and added >33 Kb of sequence (see Table 1). While the increase in contig N50 was relatively small (56 Kb), Pilon completely or partially filled 61% (156 of 256) of the total captured gaps including in both homo- and hetero-zygous regions of the genome. Homozygous regions had a slightly higher fraction of completely closed gaps (33%; 8 of 24) as compared to heterozygous regions (20%; 46 of 232). Of completely filled gaps, 93% had full length alignment to the reference (including 300 bp of their flanking sequences) at 94% sequence identity or higher. Less than 100% identity is to be expected when comparing a heterozygous genome assembly against a flat reference. In several of the lower-identity cases, most of the base differences were in the flanks present in the draft assembly rather than the filled gap itself, suggesting that the gaps may have been caused by the original assembler's inability to assemble sequence through a highly polymorphic region.

Pilon also identified and corrected regions in the reference assembly where the read alignment evidence disagreed, including 44 regions that were likely mis-assembled. The nearly 27,000 corrected single-bases were mostly at heterozygous sites; Pilon identified these as potential bases to fix, as the majority of read-evidence favored an alternate allele from the reference base in the draft. These positions represented about half of the ∼70,000 heterozygous SNP positions in this *Candida* genome [23]. While we did not investigate every change that Pilon made to this assembly, our results indicate that Pilon is suitable to be run on larger diploid genomes and can improve the quality of a draft assembly, resulting in fewer and longer contigs and an improved gene set.

#### Assembly improvements in a production environment

Given promising results from the benchmarking experiments, we implemented Pilon in the Broad Institute's *de novo* genome assembly production pipeline and assessed its performance by comparing assembly metrics from Pilon-improved assemblies of 50 representatives of the *Enterobacteriaceae* (including *Escherichia, Klebsiella, and Enterobacter*) to non-Pilon improved versions. Pilon reduced the mean number of contigs in the 50 assemblies from 33.7 to 20.9 (see Figure S2), a 38% reduction in total contigs representing closure of 47% of captured gaps. As a result, Pilon nearly doubled the contig N50 from 392 Kbp to 780 Kbp (99% increase; see Figure S3), capturing, on average, an additional 14,681 bp of sequence per assembly. This increase in genome size equates roughly to the addition of ∼14 genes per genome (based on the average bacterial gene size of ∼1 kb). Scaffold numbers were unchanged since, currently, Pilon does not attempt to join or break scaffold structures.

### Variant detection evaluation

#### Assessing accuracy of polymorphism calls

To test the accuracy of Pilon's variant detection capability, we used BWA to align approximately 200-fold coverage of reads from the same *M. tuberculosis* F11 fragment and long insert libraries used in the assembly improvement assessment to the *M. tuberculosis* H37Rv finished reference genome. We generated two sets of variant calls with Pilon, one using both fragment and long insert reads as input, and one using only fragment reads. We also ran two popular variant detection tools, GATK UnifiedGenotyper (GATK-UG) and SAMtools/BCFtools (SAMtools), starting with the same aligned fragment BAM. All variant sites, including substitutions, deletions or insertions, were identified and two categories of variants were assessed: single nucleotide polymorphisms (SNPs) and multi nucleotide polymorphisms (MNPs) greater than 1 bp. Predicted polymorphisms were compared to a curated truth set of variants produced by comparing the *M. tuberculosis* F11 finished genome to the finished *M. tuberculosis* H37Rv genome (see Methods), resulting in a list of 1,325 events (summarized in Table 2) of which the majority were SNPs. We then compared Pilon's performance to that of the other two variant detection algorithms.

**Table 2:** Summary of variant types curated in the *M. tuberculosis* H37Rv and *M. tuberculosis* F11 finished genome comparison.

Type of variation between F11 and H37RV	Total found
Single substitution	1012
Single insertion	26
Single deletion	31
Multi substitution	13
Multi insertion	56
Multi deletion	47

The full list can be found in Table S8.

Overall, Pilon performed better in identifying SNPs, including single nucleotide insertions and deletions than did GATK-UG or SAMtools (Table 3). Pilon identified 8 to 11 percentage points (pp) more single nucleotide substitutions, 4 pp more single nucleotide deletions, and 4 to 8 pp more single nucleotide insertions from the curation set than did GATK-UG or SAMtools, respectively. Pilon's ability to precisely call single nucleotide substitutions was also high - only 3% of calls were not accounted for in the curation set - which was on par with the other two tools. Similarly, Pilon had perfect precision in calling single nucleotide insertions and only 5% of single nucleotide deletion calls were not accounted for in the curation set.

**Table 3:** Recall and precision metrics for *M. tuberculosis* F11 variants called against *M. tuberculosis* H37Rv by Pilon (with and without long insert library data), GATK UnifiedGenotyper and SAMtools.

	Pilon			GATK			SAMtools			Pilon-frags		
	R	P	F	R	P	F	R	P	F	R	P	F
Single substitution	0.96	0.98	**0.97**	0.85	0.98	**0.91**	0.88	0.93	**0.90**	0.94	0.98	**0.96**
Single insertion	0.83	1	**0.91**	0.75	1	**0.86**	0.79	1	**0.88**	0.79	1	**0.88**
Single deletion	0.91	0.95	**0.93**	0.87	0.9	**0.86**	0.87	1	**0.93**	0.87	0.95	**0.91**
Multi substitution	1	0.95	**0.97**	0.67	N/A	**N/A**	1	0.98	**0.99**	1	0.95	**0.97**
Multi insertion	0.63	0.73	**0.68**	0.17	0.79	**0.28**	0.21	0.5	**0.30**	0.63	0.76	**0.69**
Multi deletion	0.73	0.9	**0.81**	0.27	0.75	**0.4**	0.39	N/A	**N/A**	0.71	0.87	**0.78**

The three rows marked with 'Single' indicate single nucleotide variants. The three rows marked with 'Multi' indicate variants involving two or more nucleotides, which also include very large events that span several Kb. Recall (R) is the fraction of curated events that were called by the program. Precision (P) is the fraction of calls that the program made that were also described in the curation. The F-measure is the harmonic mean of recall and precision and provides measure of the trade-off between recall and precision. “N/A” indicates that all events of this type were captured in another variant category.

For MNPs, we allowed for a combination of two or more smaller events in the prediction set to contribute to a larger variant since there may be equivalent ways of representing the changes as a series of smaller edits (see Methods). Pilon greatly outperformed the other two variant callers in accurately identifying variants that involved more than one nucleotide (see Table 3; bottom three rows). Pilon identified three times as many multi nucleotide insertions as either GATK-UG or SAMtools (63% versus 17 or 21% of curated events), but made slightly more false predictions. For multi nucleotide deletions, Pilon identified two times as many events from the curation set than did GATK-UG or SAMtools and made fewer unsupported calls. In addition, Pilon identified all six curated multi-substitution events while the other two tools missed at least one, even when multiple SNPs were accounted for in these regions.

**Figure 4:**
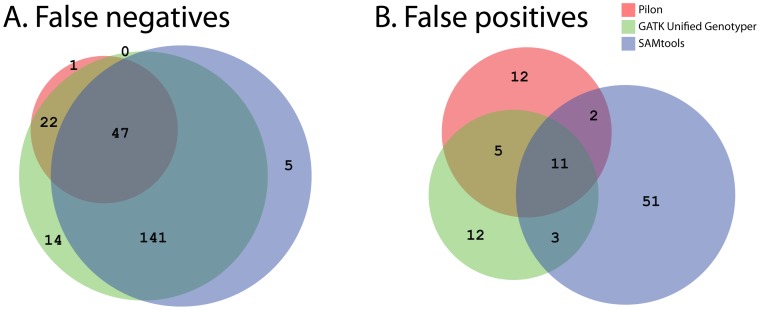
Venn diagram of the overlap in false negative (A) and false positive (B) calls by the three variant detection tools, Pilon, GATK UnifiedGenotyper and SAMtools. False negative calls are the number of unique events from the curation set that was missed by each tool. Overlaps in the Venn diagram show the number of variants that were missed by multiple tools. False positive calls are the number of predictions from *M. tuberculosis* F11 that were not supported by the curation set. Overlaps indicate predictions that were shared among tools.

We next examined how overlapping the three tools were in either missing or overcalling variants. Panel A of Figure 4 summarizes the total number of variants appearing in the curation set that could not be detected by one or more of the variant callers. Pilon uniquely missed only one curated variant, while SAMtools and GATK-UG missed many more (32 and 13, respectively). The majority of variants that were missed by Pilon were also missed by SAMtools and GATK-UG (52 events). In addition, all three tools made predictions that were not supported by the curation set (summarized in Panel B of Figure 4), but, among unique unsupported events, Pilon and GATK-UG had ∼3-fold fewer than SAMtools. Altogether, there were only 21 predictions where two or more of the tools agreed that there should be a variant called, most of which were SNPs, although four of the seven events shared by Pilon and GATK-UG were multi-nucleotide indels (5–15 nt in length).

Given the broad definition of ‘multi’ in Table 3 (>1 bp), we also evaluated how well Pilon performed for variants that were larger than 50 bp (see Table 4). We chose 50 bp since it is a length that is larger than the size of events for which short-read aligners are typically able to align, but shorter than the individual read length of the data used (101 bp). Overall, Pilon was able to accurately identify 74% of these large variants, including 100% of substitutions, 68% of insertions and 77% of deletions from the curated list. Of the eleven insertions that were missed by Pilon, eight involved a repetitive element (5 tandem insertions and 3 IS6110 insertions). Similarly, the six deletions not detected by Pilon involved deletion of one or more copies of a tandem repeat. Four of these tandem repeat regions were correctly reported by Pilon as possible tandem repeat variants in its standard output, but Pilon currently makes no attempt to provide a definitive copy number call in the presence of significant tandem repeat structures. Pilon also identified three events >50 bp that did not match variants in the curation set. These unsupported calls occurred within complex variable regions of the genome in which multiple nearby repeat structures prevented Pilon from correctly identifying the precise correct location or form of the events.

**Table 4:** Pilon's performance in calling variants in *M. tuberculosis* F11 that were larger than 50 nt.

	Pilon		Pilon-frags	
	Missed	Called	Missed	Called
**Insertion**	11	23	12	22
**Substitution**	0	5	0	5
**Deletion**	6	20	10	16

Variants are divided by type across the rows. Missed variants are those that were annotated in the curation, but were not identified by Pilon. The called variants are those that were annotated in the curation that Pilon accurately identified.

Since Pilon performed well in identifying and resolving MNPs and since GATK-UG and SAMtools were not explicitly designed to call these large variants, we sought to compare Pilon's MNP calls to that of methods specifically designed for MNP detection [11]. Though neither was described for use on microbial data, we evaluated how well BreakDancer [24] and CLEVER [25], two algorithms developed to detect large variants in eukaryotes, performed in calling MNPs on the *M. tuberculosis* test set. BreakDancer was unable to identify any MNP found in the curation set and CLEVER identified 21 multi nucleotide deletions, of which only 1 corresponded to a variant in the curated list. No large insertions or substitutions were predicted (data not shown).

#### Evaluating Pilon variant calls without long insert data

It is unsurprising that Pilon was better able to call larger variants since it is optimized to use both fragment (or small) and long (or large) insert libraries. Since many sequencing projects do not have access to long insert data and to also make a more direct comparison to existing variant callers that are not optimized to accept these data, we evaluated Pilon's performance using data from fragment insert libraries alone. To do this, we ran Pilon using the aligned fragment paired end reads from the *M. tuberculosis* F11 genome to the *M. tuberculosis* H37Rv finished reference genome. We then compared this output (“Pilon-frags”) to the previously analyzed output from GATK and SAMtools and to Pilon output using data from both library types (“Pilon”).

Pilon-frags performed well in identifying both single and multi nucleotide variants (see Table 3). Pilon-frags identified only 2 pp fewer single nucleotide substitutions, 4 pp fewer single insertions and 4 pp fewer single deletions as compared to the original Pilon output. Pilon-frag performance in calling SNPs was better or on par with both GATK-UG and SAMtools. Remarkably, Pilon-frags was also able to identify a large fraction of the MNPs, with nearly identical performance to Pilon with long insert read data. Pilon-frags also performed very well in calling variants larger than 50 bp (see Table 4), with one less insertion call and 4 fewer deletions calls as compared to Pilon.

To better understand the qualitative differences in what Pilon and Pilon-frags reported, we examined the concordance between results for each variant type. For SNP calls, we observed high concordance in the outputs from Pilon-frags and Pilon (95.2%; 871 of 915 events) (see Table S5). Discordance in SNPs often involved a position where a variant was found in both Pilon runs, but was considered high quality in one and low quality in the other. In fact, only 7 of 915 SNPs (0.8% of total) were confidently predicted to differ between the two Pilon run conditions, suggesting that the value of long insert library data when calling SNPs is small. However, for SNPs within repetitive regions of the genome, long insert data appeared to be very helpful in disambiguating these events (Table S6). Small indel variant calls were also highly concordant for the two Pilon runs (93.3%; 56 of 60 events), and 78.5% concordance (73 of 93) for large indels.

For larger variants, the discordance between Pilon with and without long insert data was larger (see Table S5), particularly in regions of the genome encoding transposable IS6110 repeat elements. While Pilon-frags detected many of these events, the sequences that were assembled and reported at these sites were often incomplete, as illustrated in Table S7. Given the length of the IS6110 repeat (∼1.3 Kbp), the fragment pairs — only ∼180 bp apart — were unable to span the entire length of the IS6110 elements, leading to two large indels being called, one coming in from each side of the IS6110 *e.g.* Table S7, position 1,541,957. Pilon's improved ability to capture the full sequence of larger insertions is the primary value of including long insert read data for variant calling applications.

#### Assessing large-scale genome duplications

In addition to identifying substitutions and indels of various sizes, Pilon is able to identify areas in which the read evidence suggests additional copies of large genomic regions (>10 Kbp) compared with the input draft assembly or reference genome. These regions could indicate large collapsed repeats in an assembly improvement application or large genomic duplications in a variant detection application. To evaluate Pilon's ability to identify large duplications, we re-sequenced *M. tuberculosis* T67, a strain previously reported to harbor a large-scale duplication [26], using fragment and long insert libraries, and aligned the reads to the *M. tuberculosis* H37Rv finished reference. Pilon was then run to detect variants in T67 using H37Rv as a reference.

Pilon identified two duplication events that were >10 Kbp in size and separated by ∼3 Kbp at *M. tuberculosis* H37Rv coordinates 3,494,063–3,551,070 (57 Kbp) and 3,554,192–3,712,284 (158 Kbp) resulting in a combined duplication of ∼215 Kbp. The left gene boundary (Rv3128c) of the first predicted duplication and right gene boundary (Rv3427c) of the second predicted duplication corresponded to the upstream and downstream boundaries in the previously reported *M. tuberculosis* T67 duplication [26]. Upon closer inspection, the 3 Kbp intervening region contained two copies of the IS6110 element, which are routinely found in multiple copies within the *M. tuberculosis* genome (16 copies in H37Rv) [27]. Because these elements occur so frequently in the genome, the incremental coverage from the duplication was not sufficient for Pilon to identify them as part of the duplication event, breaking a true large duplication into two reported pieces.

## Discussion

Pilon is an assembly improvement algorithm and variant caller that identifies differences between a draft assembly or closely related reference assembly using evidence supported by the sequencing data, resulting in either an improved assembly or a list of variants in VCF format. We have demonstrated Pilon's performance on several microbial genomes with varying GC-content and different ploidy. Our results indicate that applying Pilon yields more contiguous and accurate assemblies. Furthermore, variant calls made by Pilon are of high quality when compared with other state-of-the-art tools, and Pilon's ability to find insertion and deletion events considerably larger than read-lengths sets it apart from traditional variant calling tools.

For many of the sub tasks that Pilon performs, there are a variety of existing tools that might be used in sequence to achieve similar output as Pilon. However, using existing tools to achieve the full complement of analyses performed by Pilon would require implementation of a complicated workflow that hands data around to various tools, and development of post-processing algorithms to ensure that results from the various tools are in agreement. Pilon is a single, benchmarked tool that performs comparably well, if not better, than other tools that do only a fraction of the work. In addition, Pilon is easy to use and is successfully being utilized for assembly and variant detection of thousands of data sets in Broad Institute's production pipelines.

We showed that the assembly improvements that were introduced by Pilon were both accurate and biologically relevant. In particular, several highly repetitive genes that were captured by Pilon are known to play a role in virulence and host-pathogen interactions [28], [29]. To date, it has been difficult to study these genes comparatively, because they are often not captured or are only partially captured in draft assemblies. Furthermore, we were able to place more genes with repetitive features accurately in the genome. In the *M. tuberculosis* case, Pilon improved the sequence accuracy and placement of genes encoding transposases, which have an important role in genome reorganization in this species [27] and are used in strain typing schemes [30]. In addition, for *M. tuberculosis*, Pilon had a significant impact on the repetitive, GC-rich and not well understood PE and PPE genes, an expanded family of highly repetitive genes that account for about 10% of the gene repertoire in this species [31], and are implicated in pathogen-host interactions and virulence [32], [33]. Pilon was able to resolve these repetitive structures because of its ability to use the long-distance mate pair information afforded by long insert libraries. In addition, data from long insert libraries often enable Pilon to completely fill in large sequence insertions and assemble across gaps.

For variant calling, the primary benefit of Pilon over other variant callers is its ability to capture large sequence polymorphisms and highly polymorphic local regions by performing a local assembly to generate complete sequences. By capturing intervening sequences in large variants, Pilon enables a more comprehensive view of the biological differences between strains *e.g.*, new genes that confer antibiotic resistance or virulence. In addition, by integrating the SNP and large sequence variant detection in a single tool, Pilon is less likely to erroneously call SNPs in regions that are affected by a large variant. Long insert libraries provide information that resolves larger, multi-nucleotide events, in particular by allowing Pilon to completely assemble inserted sequences. However, even without long insert libraries, most of these events are identified, albeit often with an incomplete alternative sequence.

Pilon was also able to perform comparatively well in calling small variants, including SNPs and small indels. While all three variant callers benchmarked in this study had similar precision in their calls, Pilon demonstrated better recall (fewer false negatives) on single-base polymorphisms. There are two reasons for this. First, there are a few local areas of the genome with very high polymorphism rates between F11 and H37Rv. When the local polymorphism rate is very high, the short-read aligners are unable to produce enough good alignments for any of the tools to call the base differences from the resulting pileups. However, Pilon was able to detect some of these problem areas and reassemble them into block substitutions, correctly capturing dozens of polymorphic locations the other tools were unable to resolve. Second, the use of long insert libraries allowed Pilon to make definitive calls inside some repeat regions by heavily weighting long insert reads which were unambiguously anchored to the flanks of the repeat area, capturing additional true SNPs.

We note that GATK contains a highly sophisticated collection of tools for variant calling. However, several of its tools and their associated best practices for human variant calling are not applicable to many microbial projects, because they rely upon a database of known variant locations (such as those found at dbSNP) to perform recalibration. For microbial variant calling, the extent of variation across the microbial species under investigation is typically unknown, so no such catalog of variation is available *a priori*. There is also a fundamental difference between GATK and Pilon's approach to variant calling. GATK's UnifiedGenotyper is designed to be aggressive in detecting possible variants, relying on a user-controlled VariantFiltration step to filter out calls of lower confidence or quality to minimize false positives. Pilon, on the other hand, relies exclusively on internal heuristics to make a determination of which calls are confident. This makes Pilon easy to use “out of the box” in a highly automated environment, though it is less configurable than GATK for custom applications.

There are several areas where improvements could be made in future versions of Pilon with respect to assembly improvement and variant calling. First, it seems likely that there will be some benefit in iteratively applying Pilon in the assembly improvement and insertion variant calling process. Currently, Pilon builds out the gap/inserted sequence without re-aligning reads to build off the newly extended sequence. Using an iterative strategy has recently been used with some success by PAGIT [6]. Second, Pilon currently does not attempt to make fixes to larger structural issues within assemblies or make changes to scaffold architecture. With data from long insert libraries, it should be feasible to break and/or join scaffolds accurately. Third, tandem repeats continue to be challenging and may require a more specific approach. These regions are inherently difficult with short read data because there is no unambiguous information in the data to determine how many copies are present. This challenge is true for any *de novo* assembly; in order to resolve a tandem repeat, reads are needed that anchor into unique sequence on either side and read through the entire tandem repeat sequence. Lacking this, tandem copy numbers can only be speculated from mate-pairing information, depth of coverage calculations and library insert sizes.

Currently, Pilon is rather conservative in its corrections: (i) it uses a large cut-off to merge overlaps, and (ii) it will not attempt to resolve significant tandem repeats structures definitively. Notwithstanding the challenges encountered with tandem repeats, Pilon does an excellent job with other repetitive sequences and is able to fix many genes of known repetitive gene families and is able to fill in many transposable elements.

While we have evaluated Pilon's assembly improvements on both haploid and diploid genomes and obtained positive results for both, we acknowledge that there is still significant opportunity for future improvement in Pilon's handling of diploid genomes. Pilon could be enhanced to understand IUPAC ambiguity codes in its input genome and generate them in its output, and Pilon's heuristics for identifying insertions and deletions in diploid genomes could be improved, including its ability to recognize and report heterozygous indels. Finally, the local reassembly process could be improved to perform better in heterozygous regions. Even so, our results indicate that in its current form, Pilon is able to make valuable improvements to diploid genomes.

We have evaluated Pilon's performance using microbial genomes with finished references. However, there is no inherent limitation on the size of genomes to which Pilon can be applied. For example, we have used Pilon to improve assemblies of larger genomes, including 16 strains of the *Anopheles* genus (∼200 Mbp diploid genome), but we were unable to verify the accuracy of Pilon's improvements since these genomes have not been finished. Pilon runs within minutes on small microbial genomes and will complete overnight on larger eukaryote genomes, such as *Anopheles*, which is similar to the tools included in our benchmarking.

## Conclusion

Ultimately, Pilon has great utility and addresses an urgent need for better and more efficient methods to deal with the thousands of microbial genomes that are being produced. We have shown that Pilon performs well as compared to the state-of-the-art for both assembly improvement and variant detection, often outperforming these tools. Pilon is also unique in its user-friendly integrated approach to assembly improvement and is unique in its ability to identify large variants accurately in microbial genomes. As a recent addition to the production process for microbial genomes at Broad Institute, Pilon has been used to automatically improve the quality of over 8,000 prokaryote and eukaryote genomes prior to their submission to Genbank, and it has been used to call variants on over 6,000 genomes.

## Material and Methods

### Detailed algorithm description

#### Input requirements

Pilon requires an input genome in FASTA format and one or more BAM files containing sequencing reads aligned to the input genome. The BAM files must be sorted in coordinate order and indexed. For Illumina data, these BAM files are usually produced by an aligner such as BWA [15] or Bowtie 2 [34]. It is recommended that single best hit or random selection among equal best alignments is used as input into Pilon. Pilon can use three types of BAM files:

1. *Fragments*: paired read data of short insert size, typically <1 Kbp. Reads should be in forward-reverse (FR) orientation;
2. *Long inserts*: paired read data of longer insert size, typically >1 Kbp. Reads should be in forward-reverse (FR) orientation. Sequencing of long insert libraries that are generated using the standard Illumina mate-pair library preparation protocol typically result in reverse-forward (RF) read orientation, so they will need to be reversed in the BAM file.
3. *Unpaired*: unpaired sequencing read data.

To use Pilon with default arguments, read length should be 75 bases or longer and total sequence coverage should be 50x or greater, though deeper total coverage of >100x is beneficial. Pilon can also make use of longer reads, such as those from Sanger capillary sequencing and circular-consensus or error-corrected reads from Pacific Biosciences (PacBio) sequencing. However, Pilon is not currently tuned to the error model of raw PacBio reads, and their use may introduce false corrections.

Pilon makes extensive use of pairing information when it is available, so paired libraries are highly recommended. Pilon is capable of using paired libraries of any insert size, as it scans the BAMs to compute statistics, including insert size distribution.

#### Improving local base accuracy and identifying SNPs

Pilon improves the local base accuracy of the contigs through analysis of the read alignment information. First, Pilon parses the alignment information from the input data and summarizes the evidence from all the reads covering each base position. Alignments can be less trustworthy near the ends of reads, especially in differentiating between indels and base changes, so Pilon ignores the alignments from a small number of bases at each end of the read, which is configurable at run time. For each base position in the genome, Pilon builds a pileup structure which records both a count and a measure of the weighted evidence for each possible base (A,C,G,T) from the read alignments. The contribution of base information from each read is weighted by the base quality reported by the sequencing instrument as well as the mapping quality computed by the aligner.

When Pilon is building pileups from paired alignment data, only reads from “valid” pairs (*i.e*., those with the *PROPER_PAIR* flag set in the BAM by the aligner, indicating the reads of a pair align in proper orientation with a plausible separation) contribute evidence to the pileups. It is crucial that the PROPER_PAIR flag is set accurately by the tool that produced the BAM file. A count of non-valid alignments covering each position is also kept to help identify areas of possible mis-assembly. Pilon also keeps track of “soft clipping” in the alignments, which exclude sub-sections of a read which aligned poorly. A tally of soft-clip transitions is kept at each genomic location as another aid in identifying possible local misassemblies.

From the pileup evidence, Pilon classifies each base in the input genome into one of four categories:

1. *Confirmed*: the vast majority of evidence supports the base in the input genome;
2. *Changed*: the vast majority of evidence supports a change of the base in the input genome to another allele;
3. *Ambiguous*: the evidence supports more than one alternative at this position;
4. *Unconfirmed*: there is insufficient evidence to make a determination at this position due to insufficient depth of coverage by valid reads.

Ambiguous bases can occur for several reasons. If the genome is diploid, this is expected at heterozygous polymorphic locations. Difficult-to-sequence regions may result in a large enough fraction of sequencing errors to result in an ambiguous call. Finally, if the input genome has a smaller number of copies of a repeated genomic structure than occurs in the true genome (a “collapsed repeat”), the aligned reads may have originated from more than one instance of the repeat structure; where there are differences in the true instances of the repeat, the alignments can show mixed evidence.

Paired read information, especially information from long insert libraries which span a longer distance, is extremely valuable in helping resolve ambiguous locations due to collapsed repeats. Pairs for which one read lands inside a repeat element, but the other lands in unique anchoring sequence on the flanks of the repeat help to resolve the true base content of the repeat structure. Data from long inserts will typically have a higher alignment mapping quality than short-range fragment pairs that lie completely within the repeat because the fragment pairs may not be able to be placed uniquely among the repeats. Since Pilon uses mapping quality to weigh the evidence from each read, the long inserts can often pick the correct haplotype variations of the repeat structure.

Pilon includes corrections to single-base errors in its output genome, and optionally, it can also change ambiguous bases to the allele with the preponderance of evidence.

#### Finding and fixing small indels

While recording the base-by-base pileup evidence, Pilon also records the location and content of indels present in the alignments. Indel alignments which represent equivalent edits to the input genome may appear at different coordinates in the alignments. For instance, if the input genome has the sequence *ACCCCT*, but the read evidence suggests one of the Cs should be deleted (*ACCCT*), each individual read alignment might show a deletion at any of the four *C* coordinates. Pilon shifts alignment indels to their leftmost equivalent edit in the input genome, so that the evidence from all the equivalent edits is combined into evidence for a single event at a one location.

Pilon makes an insertion or deletion call if a majority of the valid reads support the change, though that threshold is lowered somewhat for longer events, as it is typically more difficult for aligners to identify longer indels in short read data. Called indels from the input genome are fixed in Pilon's output genome.

#### Fixing mis-assemblies, detecting large indels, and filling gaps

Pilon is capable of reassembling local regions of the genome when there is sufficient evidence from the alignments that the contiguity of the input genome does not match the sequencing data. For assembly improvement applications, this could be an indication of a local mis-assembly. For variant calling applications, this could be caused by insertions or deletions too large to be reflected in the short read alignments.

Pilon tries to identify areas of potential local read alignment discontiguity in the contigs of the input genome by employing four heuristics: (i) a large percentage of reads containing a soft-clipped alignment at a given base position, (ii) a large ratio of invalid pairs to valid pairs spanning a location, (iii) areas of extremely low coverage and (iv) rapid drops in alignment coverage over a distance on the order of a read length. Once Pilon has identified an area for local reassembly, it treats the suspicious region (which may be a single base or a larger region) as untrusted, using alignments to the trusted flanks on both sides to identify a collection of reads that might contribute evidence for the true sequence in the suspicious region.

Unpaired reads with partial alignments to the flanks are included in the collection. For paired data, Pilon identifies pairs in which one of the reads is anchored by proper alignment to one of the flanks (*e.g.*, with forward orientation on the left flank, or reverse orientation on the right flank), but whose mate is either unmapped or improperly mapped (*e.g.*, to a remote location in the genome). For fragment pairs, both reads of such pairs are included in the collection; for long inserts, only the unanchored read is included in the collection.

From the collected reads, Pilon builds a De Bruijn assembly graph (default K = 47). For each k-mer in the reads, it uses the same pileup structure to record the bases which follow that k-mer, including weighting by base quality. Then, the pileups are evaluated to determine the link(s) to the next k-mer(s); this results in either a single base call, resulting in one forward link to the next k-mer, or an ambiguous call, resulting in two links forward and a branch in the assembly graph. This process automatically prunes the assembly graph of most sequencing errors, as infrequent base differences are unlikely to present enough evidence to affect the forward links. A minimum coverage cutoff of five for each forward link also prunes the assembly graph of many false links that could appear because of sequencing errors.

Pilon then tries k-mers from the trusted flanks as starting points to walk into the untrusted region from each side, building all possible extensions with up to five branching points (2^5^ possible extensions). Tandem repeats with combined length >K cause loops in the local assembly graph, and they are detected by noting when the assembly walk reaches an already-incorporated k-mer. Pilon currently does not attempt to determine the copy number of such tandem repeats; instead, it will report the length of the repeat structure encountered in its standard output, and it will not attempt to close the two sides.

When no tandem repeat is detected, the resulting extensions from each side are combinatorially matched for possible perfect overlaps of sufficient length (2K+1) to be considered for closure. If there is exactly one such closure and it differs from the input genome, the assembled flank-to-flank sequence will replace the corresponding sequence in the input genome. Since the default k-mer size is 47, an overlap of 95 bases is required for closure.

If there are no closures or more than one possible closure, Pilon will identify a consensus extension from each flank. If an optional argument is set to allow opening of new gaps, Pilon will replace the suspicious region with the consensus extensions from each flank and create a gap between them; otherwise, it simply reports that it was unable to find a solution. These reports identify areas that an assembly analyst might wish to investigate manually.

Pilon also attempts to fill gaps between contigs in a scaffold (“captured gaps”) in the input genome. In order to fill captured gaps, Pilon employs the same local reassembly technique described above, treating the gap itself as the “untrusted” region. If there is a unique closure, the gap is filled; otherwise, consensus extensions from each flank are used to reduce the size of the gap. Pilon does not currently attempt to join or break scaffolds.

#### Large collapsed repeat (segmental duplication) detection

Pilon includes heuristics that attempt to flag areas indicative of large (>10 Kbp) collapsed repeats with respect to the input genome. These are characteristically large contiguous areas that appear to have double (or higher) read coverage compared to the rest of the genomic element being analyzed. Long insert data are excluded from this computation, as we have found long insert coverage to be far more variable across some genomes. Pilon does not attempt to fix these potentially collapsed regions, but it does report them in its standard output for further investigation.

In variant calling applications, large segmental duplications in the sequenced strain with respect to the reference have the same signature as large collapsed repeats in a draft assembly; a duplicated region of the genome will result in double the number of reads covering that sequence. Pilon's reporting of large collapsed repeat regions can be used to identify candidate segmental duplications.

#### Output files

Pilon generates a modified genome as a FASTA file, including all single-base, small indel, gap filling, mis-assembly and large-event corrections from the input genome. In the assembly improvement case, this is the improved assembly consensus. In variant detection mode, this is the reference sequence which has been edited to represent the consensus of the given sample more closely.

Pilon can optionally generate a Variant Call Format (VCF) [http://vcftools.sourceforge.net/specs.html] file, which lists copious detailed information about the base and indel evidence at every base position in the genome, including two scores regarding variant quality: the QUAL column, and a depth-normalized call quality (QD) field in the INFO column. For additional details on the VCF format, we refer to the VCF specification referred above. Changes generated by local reassembly, often triggered by larger polymorphisms in variant calling applications, are included as structural variant records (SVTYPE  =  INS and SVTYPE  =  DEL). Pilon can also, optionally, generate a “changes” file which lists the edits applied from input to output genome in tabular form, including source and destination coordinates and source and destination sequence. Finally, Pilon will optionally (with the —tracks option) output a series of visualization tracks (“bed” and “wig” files) suitable for viewing in genome browsers such as IGV [35] and GenomeView [36]. Tracks include basic metrics across the genome, such as sequence coverage and physical coverage, as well as some of the calculated metrics Pilon uses in its heuristics for finding potential areas of mis-assembly, such as percentage of valid read pairs covering every location.

Pilon's standard output also contains useful information, including coverage levels, percentage of the input genome confirmed, a summary of the changes made, as well as some specifically flagged issues which were not corrected, such as potentially large collapsed repeat regions, potential regions of mis-assembly which were not able to be corrected, and detected tandem repeats that were not resolved.

### Data generation

All sequencing data used for these experiments were generated from an Illumina HiSeq 2000 machine. For sequencing *M. tuberculosis* F11 and T67, two libraries were generated: one PCR-free 180 bp insert paired fragment library [37] and large insert 3–5 Kbp long insert library [38]. *S. pneumoniae* TIGR4 data also consisted of two libraries: one robotically size selected 180 bp insert paired fragment library [37] and a large insert 3–5 Kbp long insert library [38]. The sequencing data for *C. albicans* SC5314 was generated from three libraries: a robotically size-selected 180 bp insert paired fragment library [37], a gel-cut 4 Kbp long insert library [39], and a 40 Kb Fosill library [40]. Sequencing data were submitted to the Sequence Read Archive with identifiers: SRX347313, SRX347312, SRX105400, SRX110130, SRX347317 and SRX347316.

### Evaluation methods

#### Assembly improvement

All draft assemblies were generated using ALLPATHS-LG [41]. The draft assembly for *Mycobacterium tuberculosis* F11 utilized 100x of the 180 bp insert fragment library and 50x of the 3–5 Kb long insert library and was executed using ALLPATHS-LG v45395 utilizing the ASSISTED_PATCHING  = 2.1 parameter and the *M. tuberculosis* H37RV reference genome for assisting (GenBank accession: CP003248). The draft assembly for *S. pneumoniae* TIGR4 was created using ALLPATHS-LG v45925 with default parameters and using 100x of the 180 bp insert fragment library and 50x of the 3–5 Kb long insert library. The *C. albicans* SC5314 utilized 100x of the 180 bp insert fragment library, 100x of the gel-cut 4 Kb long insert library and 50x of the Fosill library, and was assembled with ALLPATHS-LG v39846 utilizing the ASSISTED_PATCHING and HAPLOIDIFY options with the *C. albicans* SC5314 reference sequence as a reference for assisting.

We benchmarked Pilon's ability to close gaps in the draft bacterial assemblies against two tools built for this purpose, IMAGE v2.4.1 [16] and GapFiller v1.10 [17]. The same sets of sequencing reads used as input to Pilon were used for IMAGE (fragment library only) and GapFiller (fragment and long insert libraries). IMAGE was run in the manner implemented in the PAGIT [6] example scripts: 6 iterations, one with a kmer size of 61, three with a kmer size of 49, and two with a kmer size of 41. GapFiller was run for 10 iterations with a libraries.txt file specifying a ratio *r* = 0.5 and library insert sizes computed by Pilon from the aligned bams.

To evaluate the quality of Pilon's single base and small indel corrections to the draft assemblies, we also ran iCORN v0.97 [18], the consensus sequence improvement tool in PAGIT, on the same draft assemblies using the same sets of fragment reads. iCORN was run in the manner implemented in the PAGIT example scripts, only changing the library insert size mean and range parameters. For TIGR4, we used a mean of 180 and a range of 120–300. For F11, we used a mean of 226 and a range of 100–500, since the PCR-free library preparation resulted in a wider range of insert sizes.

Fixes to the assemblies (Table S1) made by Pilon and the other assembly improvement tools were assessed by extracting the changed region of sequence in the output genome along with 300 bp flanks on each side. These extracted sequences were aligned to their respective finished reference genomes with BLASTN [42], and the accuracy of the changes was assessed by manually inspecting the alignments for accuracy, judging each fix as “Correct” or “Incorrect”. For larger block changes which resulted from local reassembly (gap filling and fixing of local mis-assemblies), a third category of “No worse” was established for situations in which: (i) the draft assembly contained a mis-assembly in the changed region, (ii) Pilon made a change attempting to fix the mis-assembly, and (iii) the fix was not entirely correct, but was no worse than the original problem.

For the assembly improvement statistics, *Bases added* was calculated by tallying bases added in locations where resulting fixes resulted in a net gain of bases during gap filling and mis-assembly correction processes, as reported in the Pilon standard output indicated by the "fix gap" or "fix break" lines.

#### Variant calling

Variant calls were made using *M. tuberculosis* H37Rv (GenBank accession: CP003248) as the reference and the *M. tuberculosis* F11 aligned read and long insert fragments as input data. From the sequenced fragment and long insert libraries, a random subset of read pairs was selected from each library to obtain an estimated 200x coverage of the *M. tuberculosis* H37Rv reference genome. Each library's reads were aligned to the *M. tuberculosis* H37Rv reference genome using BWA (version 0.5.9-r16) to generate BAM files suitable for input to the variant calling processes.

Pilon: Pilon was run with the —variant command line option, specifying the *M. tuberculosis* H37Rv reference genome and the above BAM file(s) as inputs. We evaluated two Pilon variant calling sets, one generated using both fragment and long insert library BAMs, and one using only the fragment library BAM.

GATK UnifiedGenotyper: Reads in the fragment library BAM were realigned by applying the Genome Analysis Toolkit (GATK version v3.2.2) RealignerTargetCreator and IndelRealigner tools on the fragment library aligned BAM file. Variants were then called from the realigned BAM file using UnifiedGenotyper run with the following settings: -nt 32 -A AlleleBalance -ploidy 1 -pnrm EXACT_GENERAL_PLOIDY -glm BOTH —output_mode EMIT_ALL_SITES. Low confidence variants were then filtered using VariantFiltration (VF) run with the following settings: —filterExpression "((DP-MQ0)<10) || ((MQ0/(1.0*DP))> = 0.8) || (ABHom <0.8) || (Dels >0.5)" —filterName LowConfidence.

These VariantFiltration settings filtered out variant calls at locations with less than 10 unambiguous read alignments, where 80% or more of the read depth had ambiguous mappings, where fewer than 80% of the reads supported the alternate allele, or more than half of the reads contained spanning deletions. This filter expression was based on one previously used to call variants from the European *Escherichia coli* O104:H4 outbreak [38], adjusting depth and allele balance thresholds to yield the best performance tradeoff between false negative and false positive results on these data.

SAMtools/BCFtools: The same aligned fragment library BAM file described above was used as input for variant calling using SAMtool/BCFtools v0.1.19 according to recommendations found on the SAMtools webpage (http://samtools.sourceforge.net/mpileup.shtml). samtools mpileup was used to generate pileups in bcf format, and variants were called using bcftools using the -bcg option. Finally, variants were filtered using vcfutils.pl varFilter -d 10 to filter out calls at locations where the aligned coverage was less than 10 reads. We chose the minimum depth of 10 to be consistent with the filtering used for GATK UnifiedGenotyper.

CLEVER and BreakDancer: The aligned fragment and combined fragment and long insert library described above were used as input for CLEVER v2.0rc3 and BreakDancer 1.3.6. clever —sorted —use_xa was used to generate calls for CLEVER. bam2cfg.pl -g –h was used to generate the BreakDancer config file, which was then used with breakdancer-max.

#### Curating differences between F11 and H37Rv

Differences between the finished *M. tuberculosis* F11 (GenBank accession: CP000717) and *M. tuberculosis* H37RV (GenBank accession: CP003248) references were curated by employing a banded Smith-Waterman algorithm to align syntenic regions of the two genomes. Alignments were run, separately, for each syntenic portion of the two sequences. When the alignment diverged significantly, the program was run again to pick up at the next syntenic block. The resulting alignments over syntenic regions identified coordinates of small blocks of mismatches, typically only a single-base long, but in some cases up to 289 bp. Areas where there was a significant break in synteny or where the banded Smith-Waterman alignment produced questionable results were analyzed using either Nucmer [43], ClustalW [44] or Blast2 [45] to verify the nature of the difference and to obtain more accurate coordinates. In some cases, the alignments proved too difficult to get accurate coordinates, but approximate definitions of these differences were obtained. The resulting table of differences between the two references (Table S8) has each difference annotated with most likely coordinates, with two exceptions where the variation between the strains was so high that it was impossible to know whether each difference was captured individually. The two highly variable regions corresponded to coordinates 1636857–1639600 and 3928967–3949709, which, together, account for less than 0.5% of the *M. tuberculosis* H37Rv genome. These regions were excluded from all variant analyses.

#### Variant Assessment

The resulting variant calls were compared to a manually curated set of differences between *M. tuberculosis* F11 and *M. tuberculosis* H37Rv as described above. Based on this comparison, recall and precision were calculated according to the strategy described in [46]. Briefly, recall is a measure of completeness of calls against the curated truth set; false negatives lower the recall score. Precision is a measure of the accuracy of the calls made; false positives lower the precision score. Specifically, recall  =  tp_c/(tp_c+fn) and precision  =  tp_p/(tp_p+fp), where tp_c is the number of true positive calls based on the curation set, tp_p is the number of true positive calls from the set of predicted variants, fp is the number of false calls from the set of predicted variants, and fn is the number of missed calls from the predicted variants based on the curation set. The F-measure is the harmonic mean of the recall and precision rates, providing an “overall” metric that captures tradeoff between recall and precision.

True positives in the prediction set had at least one variant site called in the curation set. For variants that affected more than a single base in the curation set (*i.e.*, multi nucleotide polymorphisms), we allowed for a combination of two or more smaller events in the prediction set to be marked as correct, since tools may call a densely polymorphic region as a block substitution rather than a series of equivalent single-base changes. For example, for the multi nucleotide substitution in the curation set, ACCGT  = > CCTGA, three SNP calls at the same location, A>C, C>T and T>A, would be counted as a true positive. In addition, predicted variants that affected more than 20 bases were allowed to match only partially with the curation set because there can be different ways to manually curate sites that vary among the *M. tuberculosis* F11 and H37Rv finished reference genomes. In particular, resolution of tandem repeats was challenging for both prediction and curation of variants since it was difficult to determine which copy of the repeat was inserted or deleted. In these cases, we counted the variant as correct if a similar event was predicted within 100 nucleotides.

## Availability

Pilon is open source software available under the GNU General Public License Version 2 (GPLv2). Pilon is written in the Scala programming language, and it makes extensive use of the open source Picard Java libraries (http://picard.sourceforge.net) for parsing BAM and FASTA files. Pilon is compiled into a single Java Archive (JAR) file which runs inside a 64-bit Java Virtual Machine environment. Binary and source distributions can be obtained from GitHub (http://github.com/broadinstitute/pilon/releases/). Results in this paper were obtained with Pilon version 1.5. A summary of all command-line options is available in Table S9.

Online documentation, as well as two example data sets to test Pilon on the same data as was used in this manuscript, are available from the web site http://broadinstitute.org/software/pilon/. We provide the *Streptococcus pneumoniae* TIGR4 data set as an assembly improvement example and the *Mtb* F11 data set as a variant calling example.

## Supporting Information

Figure S1 **Muscle alignment of TB F11 gene TFBG_12611.**
(PDF)

Figure S2 **Contig count reduction in production.**
(PDF)

Figure S3 **Contig N50 increase in 50 production assemblies.**
(PDF)

Table S1 **Assessment of gap filling and local reassembly fixes.** **b**: Comparison of assembly gap closures among Pilon, IMAGE, and GapFiller.
(PDF)

Table S2 **Assessment of base corrections by Pilon and iCORN.**
(PDF)

Table S3 **Detailed information regarding the gene based assessment of F11 assemblies.**
(XLSX)

Table S4 **Detailed information regarding the gene based assessment of TIGR4.**
(XLSX)

Table S5 **Summary of SNP, small in-dels, and large in-dels in M. tuberculosis F11 relative to H37Rv.**
(PDF)

Table S6 **Example SNPs only found with regular Pilon.**
(PDF)

Table S7 **Example IS6110 insertion element variation.**
(PDF)

Table S8 **Curation of Mtb F11.**
(XLSX)

Table S9 **Pilon Command Line Arguments.**
(PDF)
