# Supplementary material for: Pilon: An Integrated Tool for Comprehensive Microbial Variant Detection and Genome Assembly Improvement
Source: PLoS One. 2014 Nov 19;9(11):e112963. doi: 10.1371/journal.pone.0112963 (PMC4237348; doi:10.1371/journal.pone.0112963)
Supplement: Figure S1 — Muscle alignment of TB F11 gene TFBG_12611. (PDF) [file pone.0112963.s001.pdf]

Supplemental Figure 1: Muscle alignment of TB F11 gene TFBG_12611

Muscle alignment of a fraction of the M. tuberculosis F11 gene TFBG_12611, from reference coordinate 2,935,888 to 2,936,368, demonstrating that the draft assembly contained a small collapse of sequence while the Pilon-improved assembly corrected the sequence. The top line in each row represents the draft assembly, the second line is the Pilon improved version of the assembly. The dashes in rows four and five indicate the sequence that was missing in the draft assembly. It is important to note that this gene did not span a gap in the draft assembly and that it was contiguous collapsed sequence.

```
TBFG_12611_Draft               AGCGCCCTGATCGGCTACGCCCAGGGCGGCTCCGGCGGCCTCGGCGGCTTCGGCGAAAGT
TBFG_12611_PilonImproved       AGCGCCCTGATCGGCTACGCCCAGGGCGGCTCCGGCGGCCTCGGCGGCTTCGGCGAAAGT

TBFG_12611_Draft               ACCGGCGGCGACGGCGGCCTGGGCGGCGCCGGCGCTGTGCTCATCGGCACGGGCGTCGGC
TBFG_12611_PilonImproved       ACCGGCGGCGACGGCGGCCTGGGCGGCGCCGGCGCTGTGCTCATCGGCACGGGCGTCGGC

TBFG_12611_Draft               GGTTTCGGCGGCCTCGGTGGCGGCTCCAACGGCACCGGGGGCGCGGGCGGCGCGGGCGGC
TBFG_12611_PilonImproved       GGTTTCGGCGGCCTCGGTGGCGGCTCCAACGGCACCGGGGGCGCGGGCGGCGCGGGCGGC

TBFG_12611_Draft               ACGGGCGCCA--------------------------------------------------
TBFG_12611_PilonImproved       ACGGGCGCCACGCTGATCGGCCTGGGCGCCGGCGGCGGCGGCGGCATCGGCGGGTTCGCC

TBFG_12611_Draft               -------------------------------------------------------CGCTG
TBFG_12611_PilonImproved       GTCAACGTGGGCAACGGCGTCGGCGGTCTGGGCGGCCAGGGCGGCCAGGGCGCCGCGCTG

TBFG_12611_Draft               ATCGGCCTGGGCGCCGGCGGTGCCGGCGGTGCCGGCGGCGCCACAGTCGTTGGACTTGGT
TBFG_12611_PilonImproved       ATCGGCCTGGGCGCCGGCGGTGCCGGCGGTGCCGGCGGCGCCACAGTCGTTGGACTTGGT

TBFG_12611_Draft               GGCAATGGCGGTGACGGCGGTGACGGTGGCGGCCTGTTTAGTATCGGCGTCGGTGGGGAC
TBFG_12611_PilonImproved       GGCAATGGCGGTGACGGCGGTGACGGTGGCGGCCTGTTTAGTATCGGCGTCGGTGGGGAC

TBFG_12611_Draft               GGCGGCAACGCCGGCAACGGCGCCATGCCTGCCAATGGCGGCAACGGCGGCAACGCCGGG
TBFG_12611_PilonImproved       GGCGGCAACGCCGGCAACGGCGCCATGCCTGCCAATGGCGGCAACGGCGGCAACGCCGGG
```
