# Supplementary material for: Pilon: An Integrated Tool for Comprehensive Microbial Variant Detection and Genome Assembly Improvement
Source: PLoS One. 2014 Nov 19;9(11):e112963. doi: 10.1371/journal.pone.0112963 (PMC4237348; doi:10.1371/journal.pone.0112963)

Supplemental Figure 2: Contig count reduction in production

In a set of 50 Carbapenem-susceptible *Enterobacteriaceae* genomes Pilon reduced the average number of contigs by 42% compared to assemblies prior to running Pilon. The plot shows the distribution of contig counts before (blue) and after (red) running Pilon on 50 samples which are organized along the x-axis. The number of contigs ranges (y-axis) from just above 70 (left-side) to below 10 (right-side).

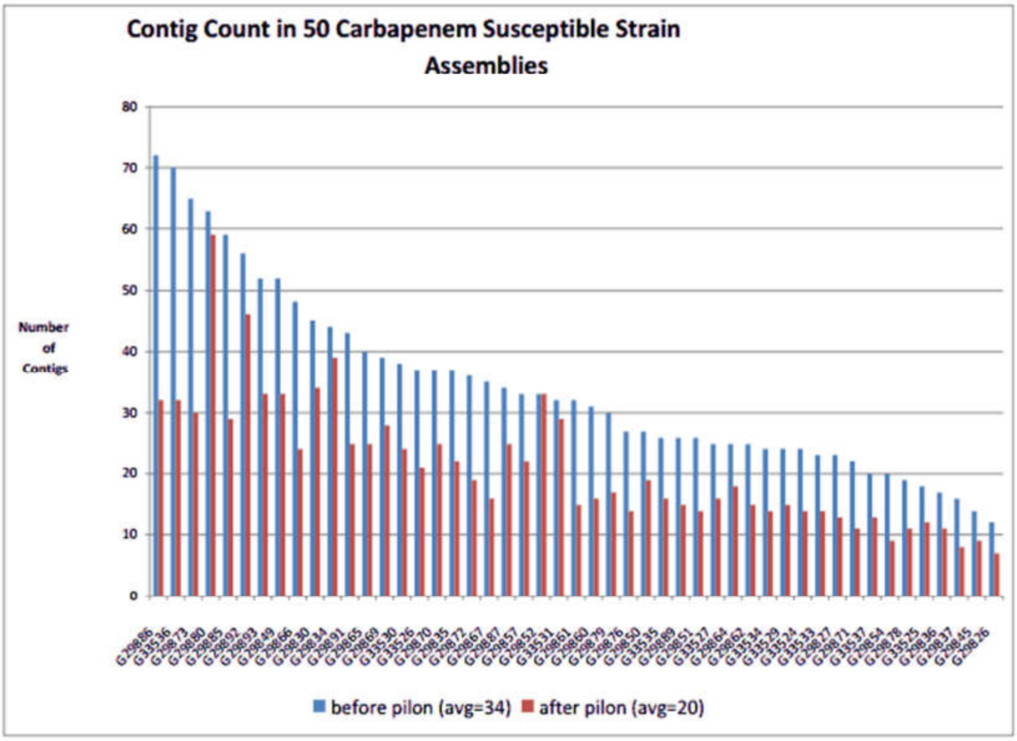

Supplement: Figure S2 — Contig count reduction in production. (PDF) [file pone.0112963.s002.pdf]
