# Supplementary material for: Pilon: An Integrated Tool for Comprehensive Microbial Variant Detection and Genome Assembly Improvement
Source: PLoS One. 2014 Nov 19;9(11):e112963. doi: 10.1371/journal.pone.0112963 (PMC4237348; doi:10.1371/journal.pone.0112963)

Supplemental Figure 3: Contig N50 increase in 50 production assemblies

Pilon is used to improve production assemblies. In a set of 50 Carbapenem-susceptible *Enterobacteriaceae* genomes, which do not benefit from reference-assisting in the assembly process, Pilon was able to increase the assembly contiguity with an average contig N50 length increase from 392 Kb to 790 Kb. The plot shows the contig N50 length for each of the 50 samples.

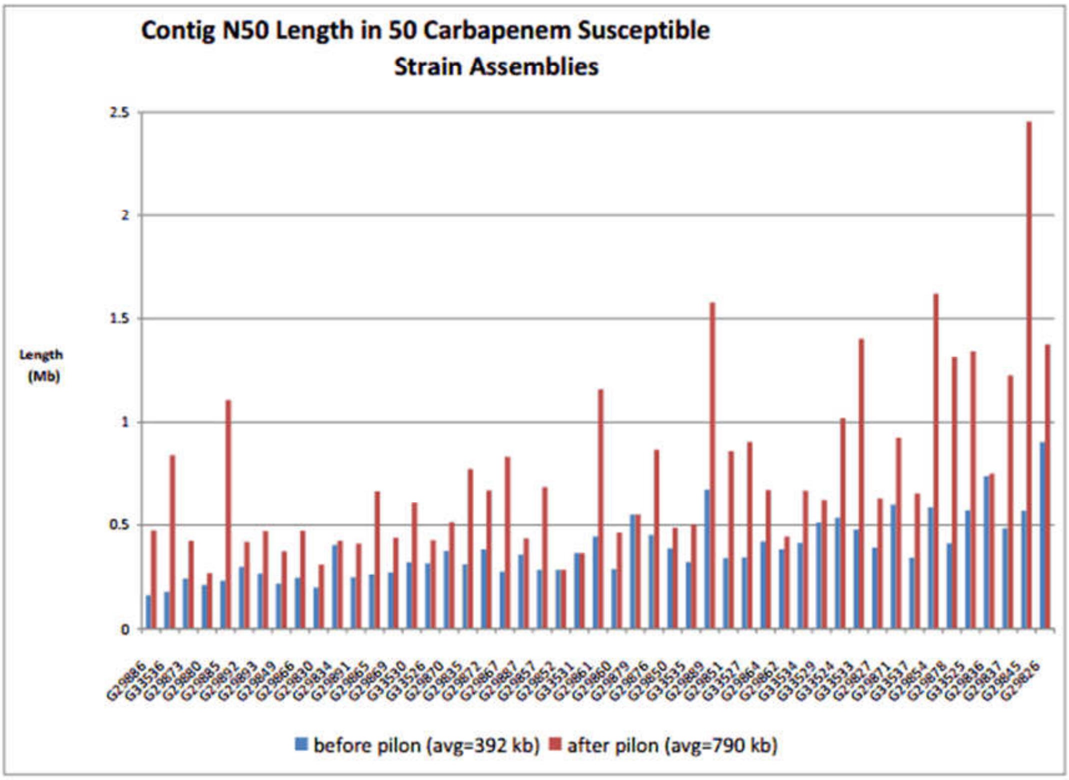

Supplement: Figure S3 — Contig N50 increase in 50 production assemblies. (PDF) [file pone.0112963.s003.pdf]
