# Supplementary material for: Pilon: An Integrated Tool for Comprehensive Microbial Variant Detection and Genome Assembly Improvement
Source: PLoS One. 2014 Nov 19;9(11):e112963. doi: 10.1371/journal.pone.0112963 (PMC4237348; doi:10.1371/journal.pone.0112963)
Supplement: Table S1 — Assessment of gap filling and local reassembly fixes. b: Comparison of assembly gap closures among Pilon, IMAGE, and GapFiller. (PDF) [file pone.0112963.s004.pdf]

Table S1: Assessment of gap filling and local reassembly fixes.

Fixes to the assemblies made by Pilon (Table S1) or IMAGE and GapFiller (Table S1b) were assessed by extracting the changed region of sequence in the output genome along with 300bp flanks on each side. These extracted sequences were aligned to their respective finished reference genomes with BLASTN, and the accuracy of the changes was assessed by manually inspecting the alignments for consistency (see Methods). In total 43% of gaps in F11 were completely closed and 76% were improved. For TIGR4, the percentages are 39% and 70% respectively. Pilon's overall closure rate was better than that of the other tools, and its accuracy was significantly higher

	**F11**			**TIGR4**		
Captured gaps	21			23		
Large changes	Correct	No worse	Incorrect	Correct	No worse	Incorrect
Complete gap closure	8	1	0	9	0	0
Partial gap closure	7	0	0	6	0	1
Discontinuity fix	2	1	0	1	0	0
Total fixes	17	2	0	16	0	1

	**F11**		**TIGR4**	
Closed Gaps	Correct	Incorrect	Correct	Incorrect
IMAGE	1	2	1	9
GapFiller	2	3	2	9
