# Supplementary material for: Pilon: An Integrated Tool for Comprehensive Microbial Variant Detection and Genome Assembly Improvement
Source: PLoS One. 2014 Nov 19;9(11):e112963. doi: 10.1371/journal.pone.0112963 (PMC4237348; doi:10.1371/journal.pone.0112963)
Supplement: Table S2 — Assessment of base corrections by Pilon and iCORN. (PDF) [file pone.0112963.s005.pdf]

Supplemental Table 2: Assessment of base corrections by Pilon and iCORN. All 47 non-ambiguous small corrections applied by Pilon were correct, whereas 35 of 49 corrections by iCORN were correct.

	**Pilon**				**iCORN**			
	**F11**		**TIGR4**		**F11**		**TIGR4**	
**Small changes**	Correct	Incorrect	Correct	Incorrect	Correct	Incorrect	Correct	Incorrect
**SNPs**	17	0	26	0	14	12	19	2
**Indels**	3	0	1	0	2	0	0	0
**Total fixes**	20	0	27	0	16	12	19	2
