# Supplementary material for: Pilon: An Integrated Tool for Comprehensive Microbial Variant Detection and Genome Assembly Improvement
Source: PLoS One. 2014 Nov 19;9(11):e112963. doi: 10.1371/journal.pone.0112963 (PMC4237348; doi:10.1371/journal.pone.0112963)
Supplement: Table S5 — Summary of SNP, small in-dels, and large in-dels in M. tuberculosis F11 relative to H37Rv. (PDF) [file pone.0112963.s008.pdf]

Supplemental Table 5: Summary of SNP, small in-dels, and large in-dels in *M. tuberculosis* F11 relative to H37Rv.

Large events are all variants that have an *SVTYPE* annotation in the VCF file. First column is the type of the event in the regular Pilon run, the second column the type of the event in the Pilon-frags run and the third column is the tally of how often the combination from the first and second column occurred.

Pilon	Pilon-frags	Count
SNP	SNP	871
SNP	Amb / LowCov / Del	14
Amb / LowCov / Del	SNP	23
SNP	REF	6
REF	SNP	1
Total SNPs found		915
Pilon	Pilon-frags	Count
Indel	Indel	56
Del	Indel	2
LowCov	Indel	1
REF	Indel	1
Total small indels found		60
Pilon	Pilon-frags	Count
Large Indel	Large Indel	73
Large Indel	-	6
-	Large Indel	14
Total large indels found		93

Legend:
Pilon: Pilon call from small (frags) and large (jump) insert libraries from *M. tuberculosis* F11.
Pilon-frags: Pilon output from only small (frags) insert library from *M. tuberculosis* F11.
REF: This base agrees with the reference.
Amb: Ambiguous; significant evidence for more than one allele at this position.
LowCov: Valid read coverage less than the threshold controlled by the --mindepth argument .
Del: This base is in a deletion or change event from another record.
