# Supplementary material for: Pilon: An Integrated Tool for Comprehensive Microbial Variant Detection and Genome Assembly Improvement
Source: PLoS One. 2014 Nov 19;9(11):e112963. doi: 10.1371/journal.pone.0112963 (PMC4237348; doi:10.1371/journal.pone.0112963)
Supplement: Table S6 — Example SNPs only found with regular Pilon. (PDF) [file pone.0112963.s009.pdf]

Supplemental Table 6: Example SNPs only found with regular Pilon.

Select example M. tuberculosis F11 SNPs found in the regular Pilon run, but not in the Pilon-frags run. Each of these examples was either an ambiguous call in the frags only run, or had low read coverage resulting in no alternative call. These examples illustrate the effectiveness of jump reads in resolving variants in repeats. In the H37Rv RVBD_0094c gene there were three SNPs found with the M. tuberculosis F11 input fragment and jump paired reads, with the jump reads mapped unambiguously. In the M. tuberculosis F11 frags-only Pilon run, the small insert reads provided ambiguous evidence with some reads calling the variant and other reads calling the reference base, and in all cases the M. tuberculosis F11 fragment reads had a low (usually zero) mapping quality. Pilon reports these three events as "Amb" variants and in the table are denoted using lowercase. In a smaller number of cases there is discordance where a SNP is found in one Pilon run but is called a reference base in the other Pilon run. Row 4 illustrates one of the four sites where the frags+jumps input Pilon run calls a SNP while the frags-only run calls a reference base. Like the SNPs in RVBD_0094c the SNP found at position 890,721 occurs in a repetitive area of the genome and the large insert jump reads allow for confident resolution of this event.

**H37Rv Position**	**H37Rv (REF)**	**Pilon**	**Pilon frags**	**F11**	**H37Rv Annotation**
104,273	T	G	G	G	103,707-104,660: RVBD_0094c hypothetical_protein
104,277	C	G	G	G	103,707-104,660: RVBD_0094c hypothetical_protein
104,280	G	C	C	C	103,707-104,660: RVBD_0094c hypothetical_protein
890,721	A	C	LowCov	C	890,391-891,485: RVBD_0797 transposase

Legend:
H37Rv Position: Coordinate in the *M. tuberculosis* H37Rv reference genome.
H37Rv (REF): Base call in the *M. tuberculosis* reference genome.
Pilon: Base call in Pilon run using input of small (frags) and large (jump) insert libraries from *M. tuberculosis* F11. Uppercase indicates a high-confidence call. Lowercase indicates an ambiguous call.
Pilon-frags: Base call Pilon run using input of small (frags) and large (jump) insert libraries from *M. tuberculosis* F11. Uppercase indicates a high-confidence call. Lowercase indicates an ambiguous call.
F11: Base call in the *M. tuberculosis* F11 reference genome. This is the known truth.
H37Rv Annotation: *M. tuberculosis* H37Rv gene, coordinates, and annotation across this position.
LowCov: Valid read coverage less than the threshold controlled by the --mindepth argument .
