# Supplementary material for: Pilon: An Integrated Tool for Comprehensive Microbial Variant Detection and Genome Assembly Improvement
Source: PLoS One. 2014 Nov 19;9(11):e112963. doi: 10.1371/journal.pone.0112963 (PMC4237348; doi:10.1371/journal.pone.0112963)
Supplement: Table S7 — Example IS6110 insertion element variation. (PDF) [file pone.0112963.s010.pdf]

**Supplemental Table 7: Example IS*6110* insertion element variation.**

Example large events found in the Pilon and Pilon-frags runs corresponding to two IS*6110* elements in the *M. tuberculosis* F11 genome. In the first example at position 1,541,957 in the H37Rv genome the Pilon run identified this 1358 base event to within just 4 bases while the Pilon-frags run identified two separate smaller events, coming from each side of the known event. In the second example at H37Rv position 2,684,042, the Pilon run has identified the insertion event to within 1 base of 1358 bases total while the Pilon-frags run has also identified a single event but only an insertion length of 361 bases with an incomplete alternative sequence.

| **Pilon** | | | **Pilon-frags** | | | **F11** | | |
|---|---|---|---|---|---|---|---|---|
| position | event details | length | position | event details | length | position | event details | length |
| 1541957 | SVTYPE=DEL; SVLEN= -1358; END= 1543315 | -1358 | 1541957 | SVTYPE=INS; SVLEN=176; END= 1541957 | 176 | 1541957 | SVTYPE=DEL; SVLEN= -1354; END= 1543311 | -1354 |
| - | - | - | 1543315 | SVTYPE=INS; SVLEN=183; END= 1543315 | 183 | - | - | - |
| 2684042 | SVTYPE=INS; SVLEN= 1358; END= 2684042 | 1358 | 2684042 | SVTYPE=INS; SVLEN=361; END= 2684042 | 361 | 2684042 | SVTYPE=INS; SVLEN= 1357; END= 2684042 | 1357 |

Legend:
Pilon: Pilon run using input of small (frags) and large (jump) insert libraries from *M. tuberculosis* F11.
Pilon-frags: Pilon run using input of small (frags) and large (jump) insert libraries from *M. tuberculosis* F11.
F11: Event details in the *M. tuberculosis* F11 reference genome. This is the known truth.
position: The start coordinate of the large event in the *M. tuberculosis* H37Rv reference genome.
event details: Type of large event (SVTYPE) ; length of large event (SVLEN); the end coordinate of the large event.
length: Length of large event found by Pilon or in the F11 known truth.
SVTYPE: Type of structural variant. DEL = Deletion. INS = Insertion.
SVLEN: Difference in length between REF and ALT alleles.
END: End position of the variant described in this record
