# Supplementary material for: Pilon: An Integrated Tool for Comprehensive Microbial Variant Detection and Genome Assembly Improvement
Source: PLoS One. 2014 Nov 19;9(11):e112963. doi: 10.1371/journal.pone.0112963 (PMC4237348; doi:10.1371/journal.pone.0112963)
Supplement: Table S9 — Pilon Command Line Arguments. (PDF) [file pone.0112963.s012.pdf]

Supplemental Table 9: Pilon Command Line Arguments.

Usage:

pilon --genome genome.fasta [--frags frags.bam] [--jumps jumps.bam] [--unpaired unpaired.bam]
[...other options...]

pilon --help for option details

INPUTS:

--genome genome.fasta
The input genome we are trying to improve, which must be the reference used
for the bam alignments. At least one of --frags or --jumps must also be given.

--frags frags.bam
A bam file consisting of fragment paired-end alignments, aligned to the --genome
argument using bwa or bowtie2. This argument may be speciified more than once.

--jumps jumps.bam
A bam file consisting of jump (mate pair) paired-end alignments, aligned to the
--genome argument using bwa or bowtie2. This argument may be specified more than once.

--unpaired unpaired.bam
A bam file consisting of unpaired alignments, aligned to the --genome argument
using bwa or bowtie2. This argument may be specified more than once.

OUTPUTS:

--output
Prefix for output files

--changes
If specified, a file listing changes in the <output>.fasta will be generated.

--vcf
If specified, a vcf file will be generated.

--tracks
This options will cause many track files (*.bed, *.wig) suitable for viewing in
IGV to be written.

CONTROL:

--variant
Sets up heuristics for variant calling, as opposed to assembly improvement;
equivalent to "--vcf --fix all,breaks".

--diploid
Sample is from diploid organism; will eventually affect calling of heterozygous SNPs

--fix fixlist

A comma-separated list of categories of issues to try to fix:
"bases": try to fix individual bases and small indels;
"amb": fix ambiguous bases in fasta output (to most likely alternative).
"gaps": try to fill gaps;
"local": try to detect and fix local misassemblies;
"all": all of the above (default);
"none": none of the above; new fasta file will not be written.
The following are experimental fix types:
"breaks": allow local reassembly to open new gaps (with "local").
"novel": assemble novel sequence from unaligned non-jump reads.

--pf
Only include reads which pass quality filtering by sequencing instrument.

--targets targetlist
Only process the specified target(s). Targets are comma-separated, and each target is a fasta element name optionally followed by a base range.
Example: "scaffold00001,scaffold00002:10000-20000" would result in processing all of scaffold00001 and coordinates 10000-20000 of scaffold00002.

--verbose
More verbose output.

--debug
Debugging output (implies verbose).

HEURISTICS:

--defaultqual qual
Assumes bases are of this quality if quals are no present in input BAMs (default 15).

--flank nbases
Controls how much of the well-aligned reads will be used; this many bases at each end of the good reads will be ignored (default 10).

--gapmargin
Closed gaps must be within this number of bases of true size to be closed (100000)

--K
k-mer size used by internal assembler (default 47).

--mindepth depth
Variants (snps and indels) will only be called if there is coverage of good pairs at this depth or more; if this value is >= 1, it is an absolute depth, if it is a fraction < 1, then minimum depth is computed by multiplying this value by the mean coverage for the region, with a minumum value of 5 (default 0.1: min depth to call is 10% of mean coverage or 5, whichever is greater).

--mingap
Minimum size for unclosed gaps (default 10)

--minqual
Minimum base quality to consider for pileups (default 0)

--nostrays

Skip making a pass through the input BAM files to identify stray pairs, that is, those pairs in which both reads are aligned but not marked valid because they have inconsistent orientation or separation. Identifying stray pairs can help fill gaps and assemble larger insertions, especially of repeat content. However, doing so sometimes consumes considerable memory.
